# Some Observations of Thiersch’s Method of Skin Grafting, Illustrated by Two Cases Shown

**Published:** 1894-04

**Authors:** William Gammon


For Texas Medical Journal.

### SOME OBSERVATIONS OF THIERSCH'S METHOD OF SKIN GRAFTING, ILLUSTRATED BY TWO CASES SHOWN.

BY WILLIAM GAMMON, M. D.

[Read before the Houston District Medical Association, February 13, 1894.]

I WISH to present to the Society, this evening, two cases illustrating Thiersch's method of skin grafting, and to speak of its usefulness in the cure of ulcers.

As all of us know, one of the most troublesome things that we meet in practice, and the bane of a hospital student's life, is the ulcer crureus, or the "sore leg" of common parlance. Although easily enough healed, or rather, it heals itself when given rest and elevation, but when healed the slightest traumatism or disturbance of circulation causes it to immediately break down and necessitate a repetition of the same process, only to be repeated again and again, as long as the patient survives. We know that the cause of this breaking down is the weakness of the tissue, which is really dependent on the poor circulation caused by the contraction of the cicatrix, which cuts off the circulation by constricting and eventually obliterating the blood vessels. We also know equally as well that the cicatrix is denser, therefore weaker, in the center, from the fact that the ulcer naturally heals from the edges and gives more time for cell proliferation of common connective tissue in the center. Knowing these things, we naturally conclude that if we find some means of covering the entire ulcer at once with epithelium, we have a cicatrix which is much thinner, and equal throughout, therefore much less liable to break down. Besides this, we gain time by having the ulcer well in a few days, whereas if left to nature the process of healing takes from a week to even months, depending, of course, on the size of the ulcer and its condition. We know, though, that if the ulcer be in a healthy condition, small bits of normal skin clipped from the healthy surface and placed on the ulcer will often grow and hasten the process of healing, thereby reducing to a certain degree the denseness of the cicatrix and the liability to recurrence. Now, it is claimed for the Thiersch graft that the whole surface of the ulcer is covered at once, and the formation of cicatricial tissue arrested, thereby giving us a much more resistant surface when healing is complete. Although the strength of the scar is primary, the rapidity of recovery comes in for its share of importance when the patient is a man whose time is valuable, or a chronic hospital case who occupies a bed which might be filled by a more interesting, if not more worthy, case.

Nor is the field of application limited to leg ulcers, but the method is quite as well adapted to ulcers of other positions and characters, as has recently been demonstrated in the treatment of lupus vulgaris. Its position in cosmetic surgery is of no little importance, especially in cases of ulcers about the face which, if allowed to heal naturally, would leave unsightly contractions. But perhaps of more importance, is when the scar is likely to cause contraction to such a degree as to impair the action of or disable a limb. Though I have had no experience in grafting in such cases, I am perfectly convinced that contraction to such degree would not take place as it would if the ulcer were allowed to heal by granulation. Scars which take on a keloid form of growth which are so prone to occur in the negro race, offer us another opportunity to make use of these grafts. In fact, I see no reason why it would not be a good procedure in true keloid, which occurs without any apparent cause.

Another condition which is quite as important, if not more so, than any other, and which we illustrate by one of the cases here to-night, is burns. We all know how slow is the healing process, and how great the contraction, in cases of burns and scalds. These cases seem to have a particular propensity to painfulness, fungoid granulation and slow healing. The discharge from the ulcer is always debilitating to the patient, according to the extent of the ulcer, and needless to say that to almost any one the presence of a discharge is far from pleasant. Now, by our Thiersch's grafts we change the painful ulcer into a condition of little more than tenderness, and under appropriate tonics the patient is soon restored to perfect health.

Before describing the operation itself, a few words concerning the preparation of the patient may not be amiss. If the ulcer be healthy, no more preparation is necessary than an antiseptic dressing; but if it be septic, it should be gotten in an aseptic condition by frequent irrigation with a 1-1000 bichloride of mercury solution, and fomentations of the same or boric acid frequently changed. The next thing to determine is, from whom to obtain the grafts. If the surface to be covered be extensive, or the patient so greatly debilitated that you fear the surface from which the grafts are to be taken would not do well, then a volunteer must be found from whom the cuticle may be obtained. Of course, in the selection of such a person one must be sure he is free from syphilis or other inoculable disease. The position from which the cuticle is to be removed is of some importance, for the sake of convenience in removal. When the ulcer is located on the skin, or, for that matter, anywhere on the ventral surface of the body, the most convenient place from which to remove the graft is the anterior aspect of the thigh. Likewise, if the ulcer be on the dorsal surface, as in the case of the burn before you this evening, the grafts may most advantageously be taken from the posterior aspect of the thigh. We have done as now suggested in both cases shown you this evening, *i. e.*, in the case of ulcer crureus the grafts were taken from the anterior aspect of the thigh, and in the case of burn of the back the grafts were taken from the posterior aspect of the thigh. The person and position from which the cuticle is to be removed having been determined, the part must be shaved and thoroughly disinfected, after which an aseptic dressing is placed thereon until the patient is in position for operation. The instruments required for the operation are very few and simple: a spatula, two pairs dissecting forceps, curette, a pair of scissors, and a scalpel, or razor, complete the essential armentarium. Thiersch has designed a razor-like knife especially adapted for this purpose, which I have never used, finding a simple scalpel all that is necessary for the purpose. The instruments having been sterilized and placed in an antiseptic solution, and hot water sponges or cotton compresses and antiseptic solution having been provided, everything is ready to begin the operation. In order to insure success, proper aseptic precautions should be maintained throughout.

The patient having been anæsthetized, the operator's most convenient position is on the side of the patient which brings him nearest to the ulcer which is to be covered, with the assistant opposite, armed with hot water compresses. The ulcer and field from which the grafts are to be taken having been exposed, the first step in the operation is to remove, with the curette, all granulations from the surface to the base of the ulcer. Next carefully pare away the edges with scissors. This having been completed, the assistant covers the entire surface with hot compresses, maintaining some pressure. While the hemorrhage is being arrested by the pressure and hot water, the operator may busy himself in cutting the grafts. If the grafts are to be removed from the thigh, the skin is drawn tight, and a narrow, almost transparent ribbon shaved off, the desired length. The ribbon of cuticle is now taken on the spatula and carefully applied to the surface of the ulcer with the assistance of the forceps, commencing at one edge, and continuing this procedure till the entire surface of the ulcer is covered. The surface may now be dusted with iodoform, a piece of oil silk or rubber tissue applied next to the skin, over which is placed gauze cotton and a bandage exerting slight pressure. The denuded surface from which the grafts have been removed should be cleansed, and dressed with a dry iodoform dressing. It is not necessary to remove the dressings for a week, unless some special symptoms, such as pain, or discharge, arise. At the end of this time, the grafts have taken, and the denuded surface has healed.

This first case illustrates very well the application of Thiersch's grafts to leg ulcers. His history, in brief, is as follows: Age, 38; no history of syphilis; an ex-grocery clerk; without occupation for the last six months. When admitted to St. Joseph's Infirmary, he had on left leg three ulcers, one about the size of a half dollar, and two about the size of a five cent nickel. On the left leg, he had one ulcer about the size of a quarter dollar. He was operated on just twelve days ago. As you now see, his ulcers are covered, and this reddish surface on the left thigh is the position from which the grafts were removed. The other man, A. S., age 43, an unemployed; while asleep by a camp fire, on December 3, '93, his clothes took fire, burning a place somewhat larger than one's hand on the right side of the back, just above the iliac crest. When admitted, the ulcer was healing, but after a little time the granulations took on an unhealthy character, and the healing almost stopped. He was operated on just fourteen days ago, and, as you see now, is perfectly well.

In both these cases, every graft applied took, and at no time were there any symptoms indicating the formation of pus, or any undue inflammation, therefore the dressings were not removed till the sixth day, when the surfaces were found to be perfectly healthy, and covered with epithelium.

I am not inclined to regard these successes as an exception, but am of the opinion that, with reasonable care, failures in these cases will be the exception rather than the rule. While this is an operation which takes some little time and care, it is likewise one which is usually successful, and gratifying to both patient and operator.

